# Generation of Mesenchymal Cell Lines Derived from Aged Donors

**DOI:** 10.3390/ijms221910667

**Published:** 2021-10-01

**Authors:** María Piñeiro-Ramil, Clara Sanjurjo-Rodríguez, Silvia Rodríguez-Fernández, Rocío Castro-Viñuelas, Tamara Hermida-Gómez, Francisco J. Blanco-García, Isaac Fuentes-Boquete, Silvia Díaz-Prado

**Affiliations:** 1 Grupo de Investigación en Terapia Celular y Medicina Regenerativa, Departamento de Fisioterapia, Medicina y Ciencias Biomédicas, Facultad de Ciencias de la Salud, Universidade da Coruña (UDC), Instituto de Investigación Biomédica de A Coruña (INIBIC), Complexo Hospitalario Universitario de A Coruña (CHUAC), Servizo Galego de Saúde (SERGAS), 15006 A Coruña, Spain; maria.pramil@udc.es (M.P.-R.); clara.sanjurjo@udc.es (C.S.-R.); s.rodriguezf@udc.es (S.R.-F.); rocio.castro@udc.es (R.C.-V.); i.fuentes@udc.es (I.F.-B.); 2 Centro de Investigaciones Científicas Avanzadas (CICA), Universidade da Coruña, 15071 A Coruña, Spain; tamara.hermida.gomez@sergas.es (T.H.-G.); fblagar@sergas.es (F.J.B.-G.); 3 Centro de Investigación Biomédica en Red de Bioingeniería, Biomateriales y Nanomedicina (CIBER-BBN), 28029 Madrid, Spain; 4 Grupo de Investigación en Reumatología (GIR), Instituto de Investigación Biomédica de A Coruña (INIBIC), Complexo Hospitalario Universitario da Coruña (UDC-CHUAC), Servizo Galego de Saúde (SERGAS), 15006 A Coruña, Spain

**Keywords:** bone repair, immortalization, mesenchymal stromal cells, cell differentiation, osteogenesis, senescence

## Abstract

**Background:** Mesenchymal stromal cells (MSCs) have the capacity for self-renewal and multi-differentiation, and for this reason they are considered a potential cellular source in regenerative medicine of cartilage and bone. However, research on this field is impaired by the predisposition of primary MSCs to senescence during culture expansion. Therefore, the aim of this study was to generate and characterize immortalized MSC (iMSC) lines from aged donors. **Methods:** Primary MSCs were immortalized by transduction of simian virus 40 large T antigen (SV40LT) and human telomerase reverse transcriptase (hTERT). Proliferation, senescence, phenotype and multi-differentiation potential of the resulting iMSC lines were analyzed. **Results:** MSCs proliferate faster than primary MSCs, overcome senescence and are phenotypically similar to primary MSCs. Nevertheless, their multi-differentiation potential is unbalanced towards the osteogenic lineage. There are no clear differences between osteoarthritis (OA) and non-OA iMSCs in terms of proliferation, senescence, phenotype or differentiation potential. **Conclusions:** Primary MSCs obtained from elderly patients can be immortalized by transduction of SV40LT and hTERT. The high osteogenic potential of iMSCs converts them into an excellent cellular source to take part in in vitro models to study bone tissue engineering.

## 1. Introduction

Injuries and diseases in cartilage and bone are the main reasons of disability for aged patients, and their prevalence is predicted to increase together with the average age of the population [1,2,3,4]. Human bone marrow-derived mesenchymal stromal cells (MSCs) are self-renewing, multipotent progenitors of osteoblasts, adipocytes and chondrocytes [5]. Thanks to their multi-differentiation potential and proliferative capacities, MSCs are potential cell candidates for cartilage and bone tissue engineering [6,7,8,9]. In addition, immortalized MSC lines can be helpful to the development of in vitro models for bone diseases and regeneration studies [10,11,12,13,14]. However, MSCs obtained from aged donors show reduced proliferation and differentiation potential and are prone to senesce in vitro, which limits their usefulness for research [6,7,8,15].

Cell senescence is caused by telomere shortening or other types of cellular stress and results in the acquisition of a senescent phenotype, characterized by enlarged cytoplasm, increased lysosomal content and senescence-associated ß-galactosidase (SA-ß-Gal) activity. Telomere shortening and the resulting chromosomal instability cause the so-called replicative senescence, while other types of cellular stress, including DNA damage or oncogenic signals, cause stress-induced premature senescence (Carnero et al., 2015; Lunyak, Amaro-Ortiz and Gaur, 2017; Schmeer et al., 2019).

During in vitro culture, the same processes that promote senescence in vivo take place: due to the propagation of the cells, telomeres are shortened, DNA damage is accumulated, and other types of cell stress are produced (Wagner et al., 2009; Medeiros Tavares Marques et al., 2017). As a result of in vitro aging, MSCs display telomere shortening, decline of their growth rate and colony forming capacity, and changes in their differentiation potential from osteogenic to adipogenic (Honoki and Tsujiuchi, 2013), termed “adipogenic switch” (Ok, Song and Hwang, 2018). The predisposition of aged MSCs to senesce in vitro may be overcome by immortalization, which can be achieved by transduction of certain genes.

Since cartilage and bone disorders like osteoarthritis (OA) are more frequent in the aged population, some efforts have been made to overcome MSCs predisposition to senescence in vitro [16,17,18,19]. Different strategies have been applied to give an unlimited growth capacity to MSCs, mainly involving the transduction of viral genes and/or human telomerase reverse transcriptase (hTERT) [16,17,18,20,21,22]. Simian virus 40 large T antigen (SV40LT), as well as other viral genes, avoids cell cycle detention by meddling with Rb- and p53-mediated pathways [23], whilst hTERT avoids telomere shortening and therefore the DNA damage-induced senescence [24,25]. It is unknown which genetic alterations are required for MSC immortalization, but it is thought that it implies the suppression of both replicative and stress-induced senescence. The transduction of MSCs with only hTERT [20,22,26,27,28], E6/E7 [20] or SV40LT [16] eventually go through senescence, while the mix of E6/E7 or SV40LT with hTERT provides efficient immortalized MSCs [18,22] and adipose-derived stem cells [29].

We previously developed a method suitable for immortalizing MSCs derived from aged donors by spinoculation of SV40LT and hTERT. In this way, we previously generated four immortalized mesenchymal cell (iMSC) lines: three derived from three hip OA patients, and one derived from a hip fracture patient without OA [30]. In this study, we generated two iMSC lines by immortalization of primary MSCs derived from two elderly patients with hip fractures and without OA. These iMSC lines were characterized and results obtained from non-OA iMSC lines were compared with OA ones.

## 2. Results

### 2.1. SV40LT and hTERT Expression in Transduced MSCs

**Figure 1:**
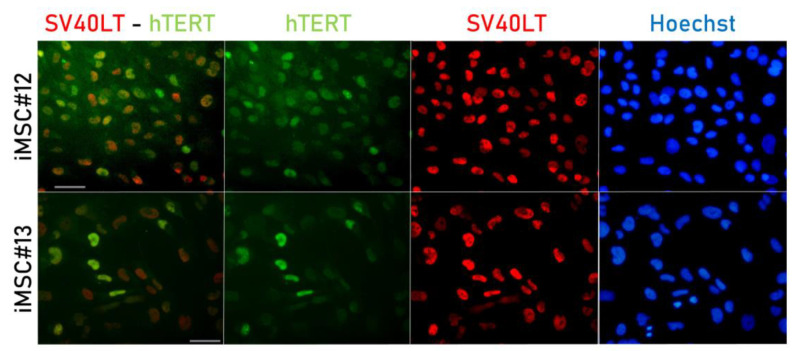
SV40LT and eGFP-hTERT immunostaining of iMSC#12 and iMSC#13; eGFP-hTERT is shown in green, SV40LT is shown in red and Hoechst staining is shown in blue. Scale bar: 50 µm.

Expression of both transgenes, SV40LT and eGFP-hTERT, was detected in both lines of transduced MSCs. Immunostaining studies allowed the detection of SV40LT by red fluorescence and eGFP-hTERT by green fluorescence. Regarding SV40LT expression, it showed a “nucleolar exclusion” arrangement, while eGFP-hTERT expression was found in both nucleoplasm and nucleoli (Figure 1). Moreover, by means of qPCR, hTERT and SV40LT expression was found in both immortalized cell lines, but not found in their primary parental cells.

### 2.2. Senescence-Associated ß-Gal Activity of Transduced MSCs

**Figure 2:**
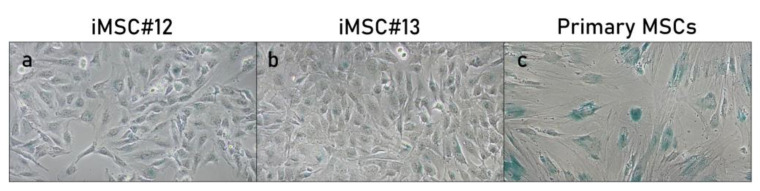
SA-ß-Gal stained iMSC#12 (**a**), iMSC#13 (**b**) and primary MSCs at the fourthpassage (**c**). Images obtained by phase contrast microscope. SA-ß-Gal activity is shown in blue. Magnification: 100×.

After more than 40 passages, both iMSC#12 and iMSC#13 showed almost no senescence-associated ß-galactosidase (SA-ß-Gal) activity (Figure 2a,b), since 2.5 ± 0.6% of the cells were ß-Gal-positive in iMSC#12 and 1.8 ± 0.3% of the cells were ß-Gal-positive in iMSC#13. Oppositely, in primary MSC#12 at the fourth passage, 57.9% of cells were positive for SA-ß-Gal activity (Figure 2c). There were no significant differences among OA and non-OA iMSCs regarding SA-ß-Gal activity (*p*-value = 0.7000).

### 2.3. Proliferative Capacity of Transduced MSCs

**Figure 3:**
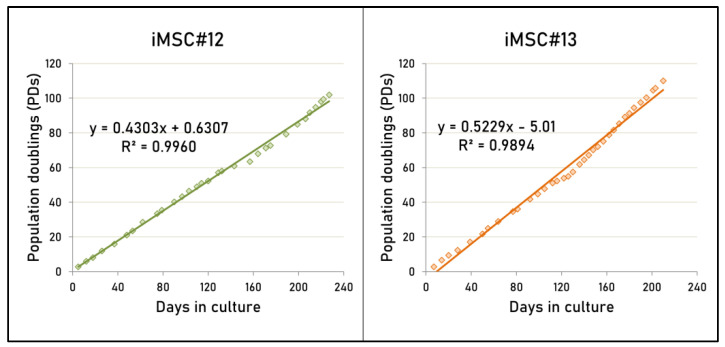
Number of population doublings (PDs) accumulated by both immortalized cell lines (iMSC#12 and #13) versus days in culture. PDs were calculated as (log *Nf* − log *Ni*)/log 2 (where *Nf* is the cell number at the end of a given subculture, *Ni* is the cell number used as inoculum to begin a given subculture, and log is the natural logarithm).

For more than half a year, iMSC#12 and iMSC#13 were cultured upon 100 population doublings (PDs). For both immortalized cell lines, a continuous growth ratio was evidenced after regression analysis (R > 0.95 and *p*-value < 0.0001) (Figure 3). Mean generation time of immortalized cell lines within the 15th and the 50th passages was 2.2 ± 0.7 days for iMSC#12 and 2.0 ± 0.7 days for iMSC#13. No significant differences were found among OA and non-OA iMSCs regarding generation time (*p*-value = 0.1345).

### 2.4. Mesenchymal Surface Marker Expression in Transduced MSCs

A phenotypical study of both transduced cell lines was developed. For this purpose, the expression levels of several mesenchymal (CD29, CD44, CD73, CD90 and CD105) and hematopoietic (CD34 and CD45) surface markers was determined in primary MSCs, SV40LT-transduced MSCs (T-MSCs) and iMSCs #12 and #13. In all cases, more than 93% of the cells analyzed were positive for CD29, CD44 and CD90. For CD73, T-MSCs showed a 58.7% of expression, which was lower than its expression in primary and immortalized, hTERT-transduced MSCs. CD105 expression increased from primary MSCs to iMSCs, but it decreased in late- versus early passage immortalized cell lines. In all cases, cell positivity for hematopoietic markers (CD34 and CD45) was 2.1% or less (Table 1).

### 2.5. Multipotency of Transduced MSCs

To find out if transduced cells showed multi-differentiation potential, primary MSCs, T-MSCs and iMSCs were grown on osteogenic, adipogenic and chondrogenic differentiation medium. The ability for multilineage differentiation towards the specific lineages was assessed by molecular and histological studies.

#### 2.5.1. Histological Analysis

**Figure 4:**
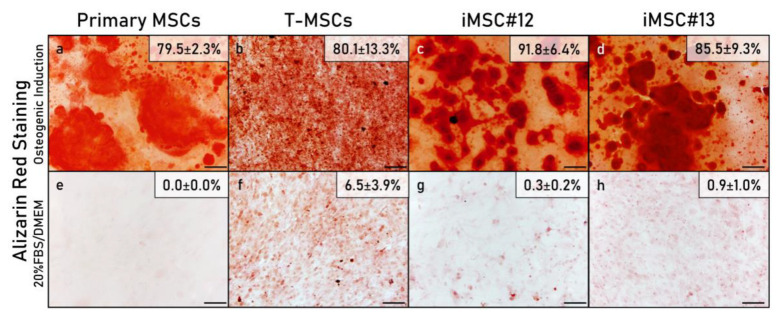
Mineralization analysis by means of Alizarin Red staining of primary MSCs, T-MSCs and iMSCs #12 and #13 after 3 weeks cultured in osteogenic medium (**a**–**d**) or grown in control medium (20%FBS/DMEM) (**e**–**h**). For each sample, the average percentage of the area stained by Alizarin Red is indicated. Information from primary parental MSC#13 and T-MSC#13 was not acquired because of cell number restrictions, and therefore only primary MSC#12 and T-MSC#12 are shown. Scale bar: 100 µm.

Primary MSCs, T-MSCs and iMSCs retained osteogenic potential since they were capable of differentiating into osteocytes after three weeks of osteogenic induction. Their osteogenic potentials were compared by Alizarin Red staining, which stains calcium phosphate deposits in red. The mineralized area was similar between primary MSCs (4th passage) (Figure 4a) and T-MSCs (8th passage) (Figure 4b) and moderately larger in iMSCs (50th passage) (Figure 4c,d). Primary MSCs grown on control medium did not show mineralization (Figure 4e), but T-MSCs control evidenced a very slight degree of mineralization (Figure 4f). Both lines of iMSCs grown on control medium were almost negative for Alizarin Red staining (Figure 4g,h).

**Figure 5:**
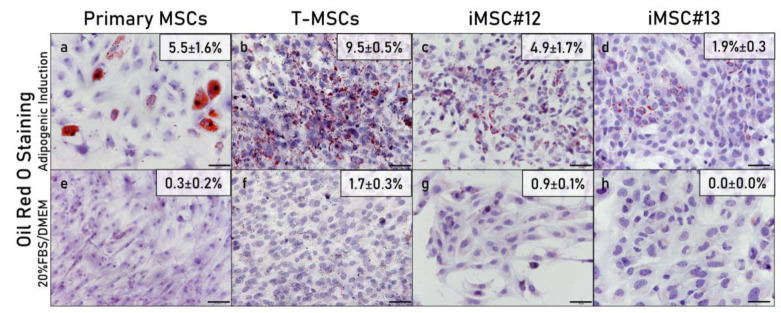
Adipogenic potential analysis of primary MSCs, T-MSCs and iMSCs #12 and #13 after 3 weeks cultured in adipogenic medium (**a**–**d**) or grown in control medium (20%FBS/DMEM) (**e**–**h**). For each sample, the average percentage of the area stained with Oil Red O is indicated. Information from primary MSC#13 and T-MSC#13 was not acquired because of cell number restrictions, and therefore only primary MSC#12 and T-MSC#12 are shown. Scale bar: 50 µm.

Primary MSCs, T-MSCs and iMSCs adipogenic potentials were compared by Oil Red O staining, which stains intracellular lipid droplets in red. Adipogenic differentiation capacity of primary MSCs, T-MSCs and iMSCs was contrasted by Oil Red O dye, which stains lipid vacuoles in red. Primary MSCs (Figure 5a) experienced weaker Oil Red O staining than T-MSCs (Figure 5b), although primary MSCs were able to form more mature adipocytes, whereas T-MSCs and iMSCs showed smaller lipid vacuoles (Figure 5b–d). Primary MSCs grown on control medium were hardly dyed with Oil Red O (Figure 5e), while T-MSCs and iMSC#12 showed some positivity (Figure 5f,g). When cultured in control medium, iMSC#13 was negative for this staining (Figure 5h).

As for chondrogenic induction, iMSC#12 did not form aggregates when submitted to the hanging drop method, and aggregates formed by iMSC#13 could not be analyzed because they were not big enough to allow for the performance of histological techniques. When submitted to pellet culture, neither iMSC#12 nor iMSC#13 was able to produce the extracellular matrix necessary to keep the integrity of the aggregates.

#### 2.5.2. Molecular Analysis

**Figure 6:**
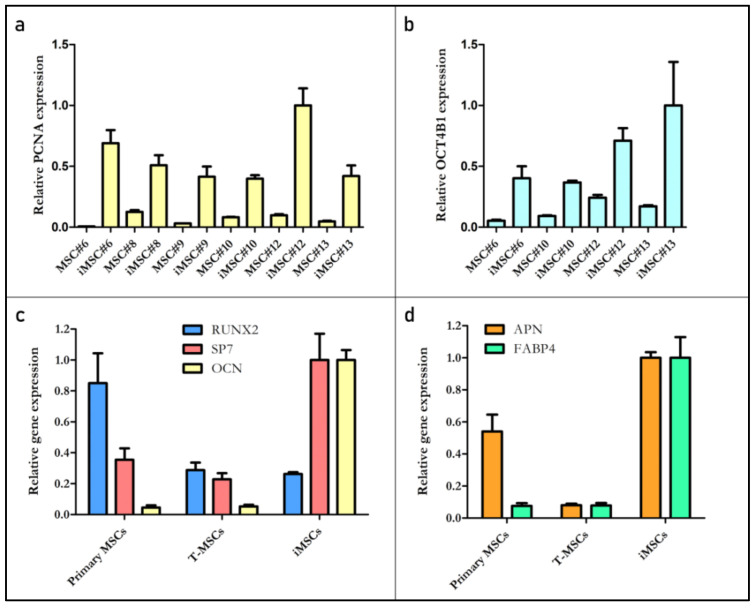
Relative expression levels (RELs) of PCNA (**a**) and OCT4B1 (**b**) in primary and immortalized undifferentiated MSCs. RELs of genes related to osteogenesis (**c**) and adipogenesis (**d**) in primary MSCs, T-MSCs and iMSCs for cell line #12. Information from primary MSC#13 and T-MSC#13 could not be obtained due to cell number limitations.

The expression of proliferating cell nuclear antigen (PCNA), which is present only in actively proliferating cells, and OCT4B1, which is related with multipotency, was analyzed in undifferentiated OA (#6, #8 and #10) and non-OA (#9, #12 and #13) iMSCs and their primary parental MSCs. The level of expression of PCNA increased in iMSCs compared with primary MSCs (*p*-value = 0.0022), but no significant differences were found between OA and non-OA iMSCs (*p*-value = 1.0000) (Figure 6a). OCT4B1 expression was also increased in iMSCs compared with primary MSCs (*p*-value = 0.0286), but, again, no significant differences were found between OA and non-OA iMSCs (*p*-value = 0.2000) (Figure 6b). Furthermore, the expression of osteogenic and adipogenic-related genes was compared in primary, T-MSCs and iMSCs in cell line #12. The highest expression of osteocalcin (OCN) was detected in iMSCs. Runt-related transcription factor 2 (Runx2) expression was higher in MSCs than in T-MSCs and iMSCs, but the highest expression of the transcription factor Sp7 expression was detected in iMSCs (Figure 6c). The highest expression of both adipogenesis-related genes was found in iMSCs. Fatty acid binding protein 4 (FABP4) was similarly expressed in MSCs and T-MSCs, while adiponectin (APN) was more expressed in primary MSCs (Figure 6d). 

**Figure 7:**
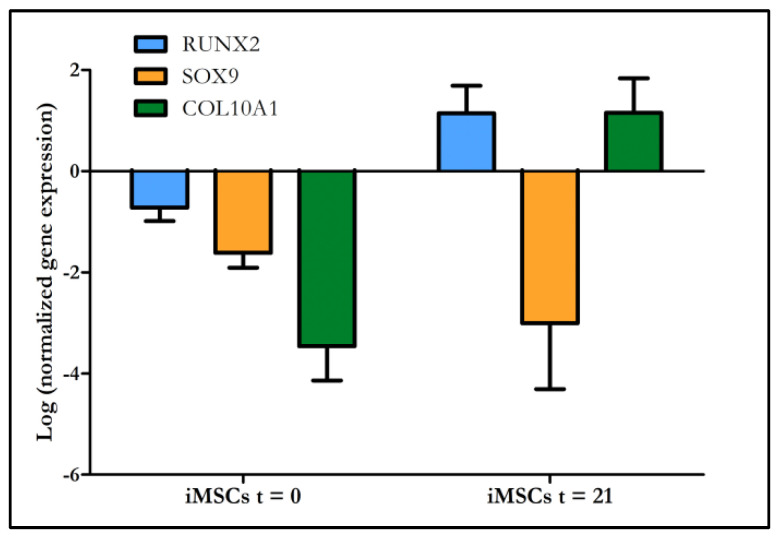
Relative expression levels (RELs) of Runx2, Sox9 and Col10A1 in iMSCs spheroids at the beginning of the experiment (t = 0) and after 21 days of chondrogenic induction (t = 21). RELs of each gene were scaled to the REL of the same gene in primary chondrocyte spheroids. Data obtained from iMSC#6, iMSC#9 and iMSC#10 spheroids were employed to calculate the mean values shown.

The expression of chondrogenesis-related genes was analyzed in spheroids formed by iMSCs (cell lines #6, #9 and #10) and primary articular chondrocytes (Figure 7). Upon chondrogenic induction, the expression of the chondrogenesis-related SRY-box transcription factor 9 (Sox9) was down-regulated in iMSCs, while the expression of the osteogenesis-related transcription factor Runx2 and type X collagen (Col10A1), a marker of chondrocyte hypertrophy, was up-regulated. In addition, the expression of Runx2 and Col10A1 was higher in iMSC spheroids than in particular chondrocyte spheroids, while the expression of Sox9 was lower. Type II collagen (Col2A1) and aggrecan expression was detected in chondrocyte spheroids but not detected in iMSC spheroids by qPCR.

### 2.6. Colony-Forming Ability and Oncogenic Potential of Immortalized MSCs

**Figure 8:**
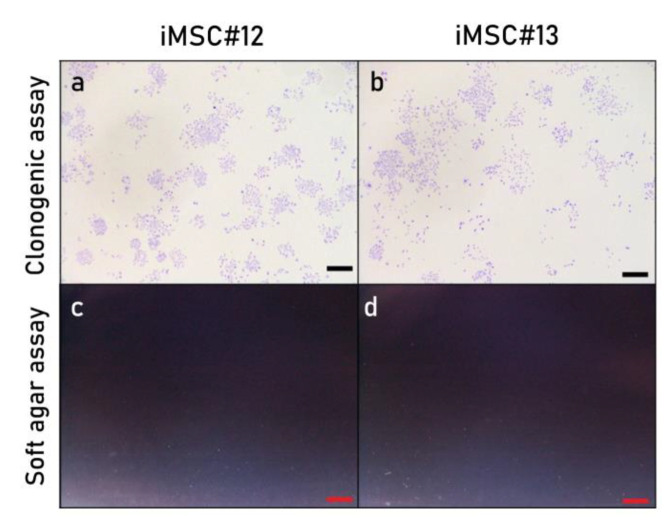
Crystal violet staining to assess the clonogenic potential of iMSC lines #12 (**a**) and #13 (**b**) and soft agar assay to assess their oncogenic potential (**c**,**d**). Scale bar: 1 mm.

A clonogenic assay and a soft agar assay were performed to assess the colony formation ability and the oncogenic potential of iMSCs. Both iMSC#12 and iMSC#13 formed colonies with wide intercellular spaces (Figure 8a,b) but were not able to grow in soft agar (Figure 8c,d).

## 3. Discussion

Although MSCs derived from aged donors are prone to senesce during in vitro culture, transduction of immortalization genes which repress both telomere shortening and p53- and Rb-mediated pathways allows them to acquire an unlimited proliferation potential [7,22,31]. The limitations associated with primary MSCs have led to the generation of several immortalized MSCs lines [16,17,18,21] which have many in vitro applications, such as the development of models of disease [13,14] and the assay of scaffolds for cartilage and bone regeneration [10,11,12].

In this study, primary MSCs derived from aged donors were immortalized by sequential spinoculation of SV40LT and hTERT, as previously described [30]. Immortalized MSCs overcame senescence and acquired an unlimited proliferation potential while maintaining most of the characteristics of MSCs. Both iMSC lines generated in this study showed high proliferation rates, unlike late-passage primary MSCs derived from aged donors. This fast proliferation may be due to the combination of SV40LT and hTERT transduction, which exerts a synergistic effect over MSC growth rate [29,32].

In addition, both iMSC lines showed high expression of the mesenchymal surface markers CD29, CD44, CD73, CD90 and CD105. However, the expression of CD73 was lower in T-MSCs. The expression of CD73 can be reduced after several passages, as has been described by other authors [33], and the loss of its expression can be accompanied by a decrease in cell growth rate. Accordingly, the reduced expression of CD73 in T-MSCs could be related to the aging of these cells, which are lacking a mechanism to repress telomere shortening and, therefore, are not completely immortalized.

Moreover, the expression of CD105 was higher in iMSCs and T-MSCs than in primary MSCs. We have previously observed that CD105 can either reduce or increase its expression from primary to immortalized cells [30], and its expression can also be reduced with passaging [29,33,34,35,36]. The role that CD105 expression plays in MSCs is still unclear [37]. There are two isoforms of CD105: L-endoglin, which is related to tumour development and angiogenesis, and S-endoglin, which has been described to supress tumour invasion and to have an anti-angiogenic effect [38,39]. The expression of S-endoglin could thus be related to the resistance of MSCs to undergo malignant transformation [40], and the loss of CD105 could be related to the acquisition of oncogenic potential. Taking together the results shown here and those from our previous study [30], only two out of six iMSC lines, iMSC#8 and iMSC#9, showed low levels of CD105, and these two cell lines were the only ones able to grow independently of anchorage in a soft agar assay.

The expression levels of genes associated with stemness are known to be altered after the transduction with immortalization genes [41]. One of these genes is OCT4, whose splice variant OCT4B1 is related to the maintenance of an undifferentiated state in both pluripotent stem cells and human somatic cells [42,43,44]. In primary MSCs, OCT4 has been related to higher proliferation and potency, and its level of expression gradually decreases as the number of passages increases [45]. In this study, iMSCs expressed higher levels of OCT4B1 than their primary parental cells, which could indicate a higher level of stemness.

One essential characteristic of MSCs is their multi-differentiation potential into osteoblasts, chondroblastas and adipocytes [5,46,47], which may be altered by immortalization [30]. Osteogenesis is the default differentiation pathway for MSCs [5,21,48], and immortalized MSCs are able to form bone both in vitro [49,50,51] and in vivo [21,52,53]. The osteogenic potential of immortalized MSCs is usually equal or higher than that of primary MSCs [16,54], while their chondrogenic potential is generally poor [17,21,26,55]. Immortalized MSCs and osteoprogenitor cells have often been reported to be able to differentiate into adipocytes [49,56,57,58,59], but adipogenic potential may be reduced after immortalization [26,60,61].

In this study, iMSCs showed higher osteogenic potential than their primary parental T-MSCs and MSCs, with stronger Alizarin Red staining and higher OCN expression. In the study by Tsai et al. (2010), the hTERT- and E6/E7-transduced immortalized MSC line 3a6 was also more osteogenic than the their parental KP cells, which were transduced with E6/E7 only. On the other hand, hTERT-transduced but not immortalized MSCs have been found to have a reduced osteogenic potential in comparison with their primary parental cells [62]. In cell line #12, iMSCs expressed the highest levels of Sp7 and OCN but lower levels of Runx2 than primary MSCs. Runx2 is a transcription factor required for the commitment of MSCs to pre-osteoblasts, while subsequent OCN production and differentiation into mature osteoblasts is regulated by a concerted action of Runx2 and Sp7. Sp7 is, at the same time, a downstream target of Runx2 [63]. Poor mineralization combined with high levels of Runx2 and low levels of OCN may indicate an immature state of the osteoblasts derived from primary MSCs and T-MSCs, which suggests that immortalization could be beneficial for the bone-forming ability of MSCs.

Both iMSC lines generated in this study were able to differentiate into the adipogenic lineage to some degree, but they did not give rise to mature adipocytes, as we had already observed [30]. Unlike iMSCs and T-MSCs, primary MSCs did form more mature adipocytes, as evidenced by Oil Red O staining. MSCs have been described to become more adipogenic during in vitro aging [64], but the adipogenic potential of immortalized MSCs has been described to be progressively reduced throughout in vitro expansions [22,60]. However, iMSC#12 expressed higher levels of the adipogenic markers APN and FABP4 than their primary parental cells upon adipogenic induction. APN and FABP4 are responsible for the formation of mature adipocytes [65], and their up-regulation upon adipogenic induction demonstrates the potential of iMSCs to differentiate into this cell lineage, although longer exposure to adipogenic stimuli may be needed to generate mature adipocytes. 

Unfortunately, the chondrogenic potential of the non-OA iMSC lines generated in this study could not be evaluated due to their inability to form three-dimensional aggregates or the small size of the aggregates after 21 days of chondrogenic induction. Nonetheless, other authors have described that immortalized MSCs have the same predisposition to hypertrophy than primary MSCs [48], with type X collagen expression [21,55] and low-quality cartilage production [66]. Gene expression analysis in previously generated iMSCs shows that these cells up-regulate the osteogenic transcription factor Runx2 and the hypertrophic marker type X collagen upon chondrogenic induction. It has been proposed that the low chondrogenic potential of MSCs is due to their committal to the endochondral ossification pathway [48].

This shift in the differentiation potential of MSCs after immortalization can be a result of either transduction or passaging. In addition, the differences found among the multi-differentiation abilities of MSCs, T-MSCs and iMSCs could be derived from the selection of cells during these processes. Extensive passaging leads to the selection of the cells with the highest growth rates in polyclonal cultures [67]. Since MSCs are heterogeneous cell populations, this arbitrary selection of cells will alter their properties, including their multi-differentiation potential.

Both iMSC lines generated in this study were non-tumorigenic. In general, MSCs have been described to be resistant to malignant transformation [34,68] and are able to obtain an unlimited proliferation potential without aberrant growth control or oncogenic features [28,34,52,54]. Other authors have also investigated the oncogenic potential of immortalized MSCs in animal models, demonstrating that only immortalized MSCs transduced with an additional proto-oncogene [34] or sub-cultured at low densities for more than 200 PDs [36] are capable of forming tumours in immunodeficient mice.

In summary, this study has shown that primary MSCs derived from aged donors can be immortalized by sequential spinoculation of SV40LT and hTERT, and that non-OA iMSCs are not different from OA iMSCs in terms of proliferation, senescence, surface makers expression and multi-differentiation potential. The high osteogenic potential of these cells makes them ideal candidates to form part of in vitro tissue engineering models for bone disease and regeneration studies.

## 4. Materials and Methods

### 4.1. Primary MSCs Isolation and Culture

The regional ethics committee of research from A Coruña-Ferrol (Spain, 2016/588) approved this study. Bone marrow-derived MSCs were isolated from two male donors (aged 88 and 95 years) with hip fractures who underwent orthopedic surgery and gave written informed consent. Bone marrow cells were obtained as previously described [30,69] and grown in Dulbecco’s modified Eagle’s medium (DMEM; Lonza, Madrid, Spain) with 20% foetal bovine serum (FBS, Gibco, Thermo Fisher Scientific, Madrid, Spain) and 1% penicillin/streptomycin (P/S, Gibco) (20%FBS/DMEM). At 80–90% confluence cells were sub-cultured with 0.1% trypsin-EDTA (Gibco). A 15-min pre-plating technique [70] was employed in the first and second passages. 

### 4.2. Primary MSCs Immortalization

Immortalization of primary MSCs was performed as described elsewhere [30]. Primary MSCs (third passage) were transduced by spinoculation using retrovirus produced by Phoenix Amphotropic cells (ATCC CRL-3213, φNX-A) [71]. Primary MSCs were transduced with SV40LT, and SV40LT-transduced cells were subsequently transduced with hTERT. φNX-A cells were transfected by employing two plasmids obtained from Addgene: pBABE-hygro-eGFP-hTERT (Addgene plasmid #28169), deposited by Kathleen Collins [72], and pBABE-puro-SV40LT (Addgene plasmid #13970), deposited by Thomas Roberts [73]. Plasmid DNA was diluted in with Opti-MEM (Gibco), and X-tremeGENE HP DNA Transfection Reagent (Roche, Sigma-Aldrich Química S.A., Madrid, Spain) was added (3 µL per µg of plasmid DNA). 

After 24 h-incubation at 37 °C, the culture medium was changed, and the transfected φNX-A cells were incubated during 48 h at 32 °C for retrovirus production. Retroviral supernatants were then filtered using a 0.45 µm filter (Millipore, Burlington, MA, USA), and hexadimethrine bromide (Sigma-Aldrich Química S.A.) was added to the retroviral supernatant (8 µg/mL). MSCs were subjected to spinoculation at 800× *g* and 32 °C for 45 min, and thereafter incubated at 37 °C for 4 h. Then, fresh culture medium with 2 mM valproic acid (Cayman Chemical Company, Ann Arbor, MI, USA) was added to the cells to induce transgene expression. After 72 h, fresh culture medium containing 2.5 µg/mL puromycin (Thermo Fisher Scientific) or 75 µg/mL hygromycin (AMRESCO, VWR International) was added for selection of transduced MSCs. Transduced MSCs were subsequently grown and sub-cultured just as primary MSCs.

### 4.3. Immunofluorescence of SV40LT and hTERT

In order to test the expression of SV40LT and hTERT, transduced MSCs were seeded in 8-well chamber slides (Millipore), fixed with 4% paraformaldehyde and permeabilized with 0.5% Triton X-100 (both from Sigma-Aldrich Química S.A.). Two primary antibodies were incubated at 4 °C overnight: rabbit anti-GFP labelled with Alexa Fluor 488 dye (A-21311; 1:500; Invitrogen, Thermo Fisher Scientific) and mouse anti-SV40LT (SV40LT clone Pab 108; 1:100; Santa Cruz Biotechnology, Dallas, TX, USA).

After that, cells were washed with phosphate-buffered saline (PBS; Dako, Agilent Technologies Spain S.L., Barcelona, Spain) and incubated at room temperature for one hour with a secondary goat anti-mouse antibody labelled with Alexa Fluor 594 dye (A-11032; 1:1000; Invitrogen). Thereafter, cells were stained with Hoechst (bisBenzimide H 33342 trihydrochloride, Sigma-Aldrich Química S.A.) and slides were mounted with Glycergel (Dako). The Olympus BX61 fluorescence microscope (Olympus Iberia S.A., Barcelona, Spain) and a coupled Olympus DP70 digital camera (Olympus Iberia S.A.) were used to obtain thefluorescence micrographs, using the cellSens Dimension software (Olympus Iberia S.A.).

### 4.4. Senescence Activity

Senescence was tested in both cell lines of MSCs using the Senescence Cells Histochemical Staining kit (Sigma-Aldrich Química S.A.) for cytochemical staining of SA-ß-Gal activity. All staining was performed after 100 PDs and at three different passages. Images of the cells were obtained with a Nikon Eclipse TS100 inverted microscope and a XM Full HD digital camera after 16 h of incubation. Randomly, ten microscope fields were counted for SA-ß-Gal-positive and negative cells to calculate the percentage of senescent cells, and the results are shown as mean percentage ± standard error. As a control, primary fourth passage MSCs were used.

### 4.5. Proliferation Analysis

The formula in Equation (1) [74] was used to calculate the proliferation of the transduced cells as cumulative PDs. At each passage, cells were detached and counted to obtain the initial and final cell numbers. The number of PDs per day was calculated at each passage to obtain the generation time for each cell line. Regression was used to analyze proliferation rates of both cell lines.

(1) $$PD=\frac{\log Nf-\log Ni}{\log2}$$

Equation (1). Formula employed to calculate population doubling (PD) at each passage, where *Nf* is the final cell number, *Ni* is the initial cell number, and log is the natural logarithm.

### 4.6. Flow Cytometric Analysis

Flow cytometry was used to analyze the expression of CD29, CD44, CD73, CD90 and CD105, which are surface markers characteristic of MSCs, as well as the expression of CD34 and CD45, which are hematopoietic stem cells markers. After being split with 0.1% trypsin-EDTA, cells were washed twice in Fluorescence Activated Cell Sorting (FACS) buffer (BD Biosciences, Madrid, Spain). Incubation with fluorescent-labelled antibodies and isotype controls (Table 2) was performed at 4 °C for 45 min. After washing, cells were resuspended in FACS buffer and transferred to polypropylene tubes (NUNC, VWR International) for data acquisition with the BD FACSCalibur flow cytometer (BD Biosciences). Obtained data was analyzed using BD Cell-Quest Pro software (BD Biosciences). 10^5^ cell events were acquired as a minimum for each assay. Results were shown as percentage of positive cells. 

### 4.7. Cell Differentiatin Induction

Multi-differentiation potential of primary and transduced MSCs was evaluated through osteogenic, adipogenic and chondrogenic cell differentiation assays. For osteogenic and adipogenic cell differentiation experiments, 2 × 10^4^ cells were seeded on 8-well chamber slides to perform histological analysis, while 10^5^ cells were seeded on 6-well plates for molecular analysis. Experiments were maintained for 21 days in hMSC Ostegenic Differentiation Medium (Lonza) or StemPro Adipogenesis Differentiation Kit (Gibco) and 20%FBS/DMEM as a control. 

For chondrogenic cell differentiation experiments, three-dimensional cell culture was chosen [75]. Both hanging drop method and pellet cultures were employed to create cell aggregates, which were subsequently incubated in hMSC Chondrogenic Differentiation Medium supplemented with 10 ng/mL of human transforming growth factor β-3 (TGF-β3) (ProSpec-Tany TechnoGene, Rejovot, Israel) for 21 days. Cell aggregates were also incubated for the same time in 20%FBS/DMEM as a control. Articular chondrocytes obtained from one donor with hip OA who underwent orthopedic surgery (female, 81 years) as described elsewhere [76] were submitted to the same differentiation protocol, and chondrocyte spheroids were employed as a positive control of chondrogenesis.

### 4.8. Histological Analysis

After osteogenic and adipogenic induction, cells on chamber slides were fixed with 4% paraformaldehyde. For osteogenic differentiation, cells were stained with Alizarin Red, and slides were mounted with DPX mounting medium (Surgipath, Leica Microsistemas S.L., Barcelona, Spain). For adipogenic differentiation, cells were stained with Oil Red O, and slides were mounted with Glycergel aqueous mounting medium. Chondrogenically-differentiated cell aggregates were fixed with 3.7% formaldehyde (Panreac Química S.L.U., Barcelona, Spain), embedded in paraffin (Merck Millipore, Merck KGaA, Darmstadt, Germany) and cut in a microtome.

Micrographs of slides were taken with the Olympus DP70 digital camera coupled to the Olympus BX61 microscope and using the cellSens Dimension software. Stained areas and staining intensity were evaluated quantitatively employing the ImageJ software (National Institutes of Health, Bethesda, MD, USA). To measure staining intensity, optical density (OD) was calculated using the formula OD = log (max intensity/mean intensity), where log is the natural logarithm. An average of optical density and percentage of staining was obtained from four different areas of each sample.

### 4.9. Molecular Analysis

RNA was isolated with TRIzol Reagent (Thermo Fisher Scientific) and chloroform (Sigma-Aldrich Química S.A.), followed by an RNA precipitation step with isopropanol (Sigma-Aldrich Química S.A.) and a washing step with ethanol (Sigma-Aldrich Química S.A.). A reverse transcription-polymerase chain reaction (RT-PCR) was performed using the SuperScript VILO cDNA Synthesis kit, following the instructions of the kit (Thermo Fisher Scientific), in an Applied Biosystems Veriti 96-Well Thermal Cycler (Thermo Fisher Scientific). The RT-PCR employed program was 10 min at 25 °C, 120 min at 42 °C and 5 min at 85 °C. Retrotranscription was performed with 2 µg of RNA when available, and obtained cDNA was diluted 1:100; otherwise, the total amount of isolated RNA was retrotranscribed and cDNA was diluted accordingly. Quantitative real-time polymerase chain reaction (qPCR) was performed in a LightCycler 480 Instrument (Roche), using LightCycler 480 SYBR Green I Master (Roche) and adding the specific primers for the genes shown in Table 3. The program of qPCR consisted of 10 min of incubation at 95 °C, 35–45 amplification cycles (10 s at 95 °C, 5 s at 60 °C and 10 s at 70 °C), one melting cycle (5 s at 95 °C, 1 min at 65 °C and up to 97 °C at 0.03 °C/s) and 20 s of cooling at 40 °C.

qPCR analysis was performed using the LightCycler 480 Relative Quantification software (Roche). Relative gene expression levels (RELs) were calculated using the qbase+ software (Biogazelle, Zwijnaarde, Belgium). RELs of osteogenesis, adipogenesis, proliferation and multipotency related genes were scaled to the sample with the highest expression of each gene. RELs of chondrogenesis-related genes in iMSC spheroids were scaled to chondrocyte spheroids. Results are expressed as mean ± standard error. YWHAZ was the most stable of the candidate reference genes tested, as determined by geNorm [77], and therefore it was employed as the reference gene in qPCR assays. 

### 4.10. Colony Formation Assay

In order to assess the colony formation ability of transduced MSCs, 5 × 10^2^ of these cells were seeded in 6-well culture dishes and grown for one week. After this time, transduced MSCs were washed with PBS, fixed with 4% paraformaldehyde and stained with 0.1% crystal violet (Sigma-Aldrich Química S.A.). Stained colonies were visualized with the Nikon SMZ 745T stereomicroscope, and pictures of the colonies were taken with a coupled Nikon DS-Fi2 digital camera (Nikon Instruments Europe B.V.).

### 4.11. Soft Agar Assay

Oncogenic potential of transduced MSCs was studied with the soft agar colony formation assay [78]. For each cell line, 3.75 × 10^3^ cells/well were inoculated in 0.375% agar (Sigma-Aldrich Química S.A.), layered on top of a 0.5% agar layer in 12-well culture dishes (Costar Corning Incorporated) and incubated for 14 days. The resultant colonies were visualized and photographed using a Nikon SMZ 745T stereomicroscope coupled to a Nikon DS-Fi2 digital camera.

## Figures and Tables

**Table 1:** Phenotypical analysis to determine the mesenchymal and hematopoietic surface markers’ expression pattern in primary MSCs, T-MSCs and iMSCs at different (early and late) passages. Passage is represented as the number of subcultures as (1) primary MSCs, (2) T-MSCs and (3) iMSCs. Data from primary MSC#13 and T-MSC#13 could not be obtained because of limited cell number, and therefore only primary MSC#12 and T-MSC#12 are shown.

Cells	Passage	CD29	CD44	CD73	CD90	CD105	CD34	CD45
Primary MSCs	3	93.3%	95.2%	71.1%	98.5%	69.8%	0.2%	1.9%
T-MSCs	(3 + 4)	98.1%	98.9%	58.7%	98.4%	77.0%	2.1%	0.8%
iMSC#12	(3 + 4 + 12)	97.4%	96.8%	85.6%	99.5%	80.1%	0.0%	0.0%
iMSC#12 (PD > 100)	(3 + 4 + 43)	97.8%	99.3%	82.1%	96.5%	78.1%	0.7%	0.2%
iMSC#13	(3 + 3 + 13)	99.3%	99.4%	98.8%	99.9%	97.7%	0.3%	0.9%
iMSC#13 (PD > 100)	(3 + 3 + 44)	97.9%	98.2%	86.1%	98.3%	90.2%	0.5%	0.6%

**Table 2:** Isotype controls and antibodies conjugated with fluorescein isothiocyanate (FITC), phycoerythrin (PE) or PE/Cy5 used for flow cytometry.

Antibody	Specificity	Clone	Source	Dilution
FITC Mouse IgG1 Isotype Control	-	ICIG1	Immunostep	1:50
PE Mouse IgG1 Isotype Control	-	B11/6	Immunostep	1:50
PECy5 Mouse IgG1 Isotype Control	-	1F8	Abcam	2:25
PE Mouse Anti-Human CD29	Human integrin β1 (ITGB1)	VJ1/14	Immunostep	3:50
PE Mouse Anti-Human CD34	Hematopoietic progenitor cell antigen 1 (HPCA1)	581	BD Pharmingen	2:25
FITC Mouse Anti-Human CD44	Homing cellular adhesion molecule (HCAM)	IM7	BD Pharmingen	1:50
FITC Mouse Anti-Human CD45	Leukocyte common antigen (LCA)	D3/9	Immunostep	3:50
PE Mouse Anti-Human CD73	Ecto-5′-nucleotidase (NT5E)	AD2	Immunostep	3:50
PECy5 Mouse Anti-Human CD90	Thymocyte differentiation antigen 1 (Thy-1)	5E10	Immunostep	1:50
FITC Mouse Anti-Human CD105	Human Endoglin (ENG)	SN6	AbD Serotec	1:50

**Table 3:** Studied genes and respective primers employed for quantitative real-time PCR (qPCR) analysis.

Gene	Forward Primer 5′→3′	Reverse Primer 5′→3′
Tyrosine 3-monooxygenase/tryptophan 5-monooxygenase activation protein zeta (YWHAZ)	GATCCCCAATGCTTCACAAG	TGCTTGTTGTGACTGATCGAC
Homo sapiens runt related transcription factor 2 (RUNX2)	TTACTTACACCCCGCCAGTC	TATGGAGTGCTGCTGGTCTG
Homo sapiens Sp7 transcription factor (SP7)	TCCCCTGTTGCCATGGTTAT	CCACCCATTCTTCAGGAGGT
Homo sapiens bone gamma-carboxyglutamate protein (OCN)	GGCGCTACCTGTATCAATGG	TCAGCCAACTCGTCACAGTC
Homo sapiens adiponectin, C1Q and collagen domain containing (APN)	GGTGAGAAAGGAGATCCAGGT	TGCTGAGCGGTATACATAGGC
Homo sapiens fatty acid binding protein 4 (FABP4)	GGATGATAAACTGGTGGTGGA	CACAGAATGTTGTAGAGTTCAATGC
Simian virus 40 complete genome (SV40)	TGGGGAGAAGAACATGGAAG	AAATGAGCCTTGGGACTGTG
Homo sapiens telomerase reverse transcriptase (hTERT)	GCTAGTGGACCCCGAAGG	CCTCCCTGACGCTATGGTT
Homo sapiens proliferating cell nuclear antigen (PCNA)	TAGACTTTCCTCCTTCCCGC	TGCCTCCAACACCTTCTTGA
Homo sapiens POU class 5 homeobox 1 (POU5F1), transcript variant 4 (OCT4B1)	AGGGAGAGGGAGAAGATGCT	GAAGCAAAGTGAGGGAGCAC
Homo sapiens SRY-box transcription factor 9 (SOX9)	GTACCCGCACTTGCACAAC	TCGCTCTCGTTCAGAAGTCTC
Homo sapiens collagen type X alpha 1 chain (COL10A1)	CACCTTCTGCACTGCTCATC	GGCAGCATATTCTCAGATGGA
Homo sapiens collagen type II alpha 1 chain (COL2A1)	TGGTGCTAATGGCGAGAAG	CCCAGTCTCTCCACGTTCAC
Homo sapiens aggrecan (ACAN)	CGGTCTACCTCTACCCTAACCA	GAGAAGGAACCGCTGAAATG
